# Motor Dysfunctions and Neuropathology in Mouse Models of Spinocerebellar Ataxia Type 2: A Comprehensive Review

**DOI:** 10.3389/fnins.2016.00572

**Published:** 2016-12-15

**Authors:** João M. Da Conceição Alves-Cruzeiro, Liliana Mendonça, Luís Pereira de Almeida, Clévio Nóbrega

**Affiliations:** ^1^Center for Neuroscience and Cell Biology, University of CoimbraCoimbra, Portugal; ^2^Faculty of Pharmacy, University of CoimbraCoimbra, Portugal; ^3^Department of Biomedical Sciences and Medicine and Center for Biomedical Research, University of AlgarveFaro, Portugal

**Keywords:** mouse, spinocerebellar ataxia type 2, transgenic, knock-in, motor impairments, neuropathology

## Abstract

Spinocerebellar ataxia type 2 (SCA2) is an autosomal dominant ataxia caused by an expansion of CAG repeats in the exon 1 of the gene ATXN2, conferring a gain of toxic function that triggers the appearance of the disease phenotype. SCA2 is characterized by several symptoms including progressive gait ataxia and dysarthria, slow saccadic eye movements, sleep disturbances, cognitive impairments, and psychological dysfunctions such as insomnia and depression, among others. The available treatments rely on palliative care, which mitigate some of the major symptoms but ultimately fail to block the disease progression. This persistent lack of effective therapies led to the development of several models in yeast, *C. elegans, D. melanogaster*, and mice to serve as platforms for testing new therapeutic strategies and to accelerate the research on the complex disease mechanisms. In this work, we review 4 transgenic and 1 knock-in mouse that exhibit a SCA2-related phenotype and discuss their usefulness in addressing different scientific problems. The knock-in mice are extremely faithful to the human disease, with late onset of symptoms and physiological levels of mutant ataxin-2, while the other transgenic possess robust and well-characterized motor impairments and neuropathological features. Furthermore, a new BAC model of SCA2 shows promise to study the recently explored role of non-coding RNAs as a major pathogenic mechanism in this devastating disorder. Focusing on specific aspects of the behavior and neuropathology, as well as technical aspects, we provide a highly practical description and comparison of all the models with the purpose of creating a useful resource for SCA2 researchers worldwide.

## Introduction

Spinocerebellar ataxia type 2 (SCA2) belongs to a group of hereditary neurodegenerative diseases—Polyglutamine (PolyQ) diseases—such as Huntington's disease (HD), Spinal bulbar muscular atrophy (SBMA), and several spinocerebellar ataxias (SCAs) (Fan et al., 2014). These disorders share a common biological cause: an abnormal repetition of CAG triplets in the open reading frame of the causative genes. These triplet expansions encode for an expanded polyglutamine tract in the respective proteins, conferring a gain-of-function mutation that triggers the appearance of the disease phenotype (Shao and Diamond, 2007; Fan et al., 2014). In SCA2 patients, this expansion is found in the exon 1 of the gene ATXN2, with a number of CAG repeats above 31 (that can go up to 200), while the healthy individuals usually possess between 13 and 31 repetitions (Magaña et al., 2013). This neurodegenerative disorder was first known as Wadia–Swami type ataxia owed to the two Indian researchers that first reported it (Wadia and Swami, 1971; Sinha, 2004). Later on, it became known as SCA2, after the discovery of the disease *locus* in chromosome 12 (Gispert et al., 1993). The study of this disease assumes a central relevance in Hispano-American and Indian populations, since it has been demonstrated to be the most prevalent form of ataxia in Mexico, Holguín (Cuba) and eastern India (Sinha, 2004; Alonso et al., 2007; Velázquez-Pérez et al., 2011). However, the existence of large SCA2 families has also been reported in the UK (Leggo et al., 1997; Giunti et al., 1998) and Spain (Pujana et al., 1999; Infante et al., 2005), where it is probably the most common SCA, and many other countries such as Australia, Germany, Italy, and Brazil. Worldwide, it is considered the second most common form of autosomal dominant cerebellar ataxia, together with SCA6, with 15% of total cases (Lastres-Becker et al., 2008b; Magaña et al., 2013).

Before the identification of the ATXN2 mutation by three different groups (Imbert et al., 1996; Pulst et al., 1996; Sanpei et al., 1996), the diagnosis of this disease was based on observations of its major symptoms, which have little variation to the other SCAs. Thereby, SCA2 clinical manifestations include a slow and progressive gait ataxia and dysarthria accompanied by leg cramps, postural tremors, decreased muscle tone, and decreased tendon reflexes (Pulst, 2015). These most often come together with sleep disturbances and oculomotor dysfunctions such as slow saccades (Bürk et al., 1996) and, in some individuals, ophthalmoparesis (Lastres-Becker et al., 2008b; Pulst, 2015). The symptoms are commonly triggered in the fourth decade of life in an affected individual and they tend to worsen in a progressive manner until the death of the patient (typically from respiratory failure), which usually occurs within 21–25 years after the onset of the disease phenotype (Klockgether et al., 1998; Lastres-Becker et al., 2008b). In most cases, the physical manifestations come together with cognitive impairments like fronto-executive dysfunction, altered short-term memory, lack of attention (Bürk et al., 2003), and psychological dysfunctions that end up in insomnia, depression, and suicidal impulses, as it was first uncovered by Reynaldo-Armiñán (cited in Lastres-Becker et al., 2008b). A more recent work by Lo et al. (2016) included a follow-up of 64 SCA2 patients that revealed a strong prevalence of clinically relevant depression (22% of all patients) and suicidal ideation (almost 50%), similarly to other SCAs.

These symptoms are usually the result of a wide neurodegenerative process, with a severe olivopontocerebellar atrophy as its most striking characteristic, together with early degeneration in the *substantia nigra* and *basal ganglia* (Estrada et al., 1999). Other brain regions such as the cerebral frontal lobes, brainstem, cranial nerves, and spinal cord also show signs of degeneration (Estrada et al., 1999; Pang et al., 2002; Ishida et al., 2011). Interestingly, *in vivo* brain MRI of 24 SCA2 patients has also revealed a significant atrophy of regions like the pontine base, the middle cerebellar peduncles and the cerebellar hemispheres, when compared with either healthy controls or patients with SCA1 and Machado-Joseph disease (MJD/SCA3l; Bürk et al., 1996).

At the cellular level, there is a severe reduction in the number of cerebellar Purkinje cells (PCs) as well as its arborization (Estrada et al., 1999; Rüb et al., 2013). The cells of the granular layer of the *cerebellum* also suffer neuronal loss, together with the brain stem and the *substantia nigra*, which might explain the parkinsonism observed in some patients (Pulst, 2015). This is a critical point, since SCA2 is the subtype of SCA that is most commonly associated with levodopa-responsive Parkinson's disease (PD) and atypical parkinsonism worldwide (Gwinn-Hardy et al., 2000; Shan et al., 2001; Park et al., 2015). Also, recent studies have found degeneration in the *striatum, pallidum*, and even the neocortex (Estrada et al., 1999; Seidel et al., 2012). In addition to this, several groups have presented compelling evidence for the existence of nuclear and cytoplasmic inclusions, stained with anti-ataxin-2, anti-expanded PolyQ (1C2) and anti-ubiquitin antibodies, similarly to what happens in other polyQ diseases (Koyano et al., 1999; Pang et al., 2002; Ishida et al., 2011). All in all, this overall degeneration in the brains of SCA2 patients goes far beyond the purely cerebellar alterations that its name might imply and it ends up affecting the whole brain.

## Disease mechanisms of SCA2

**Figure 1:**
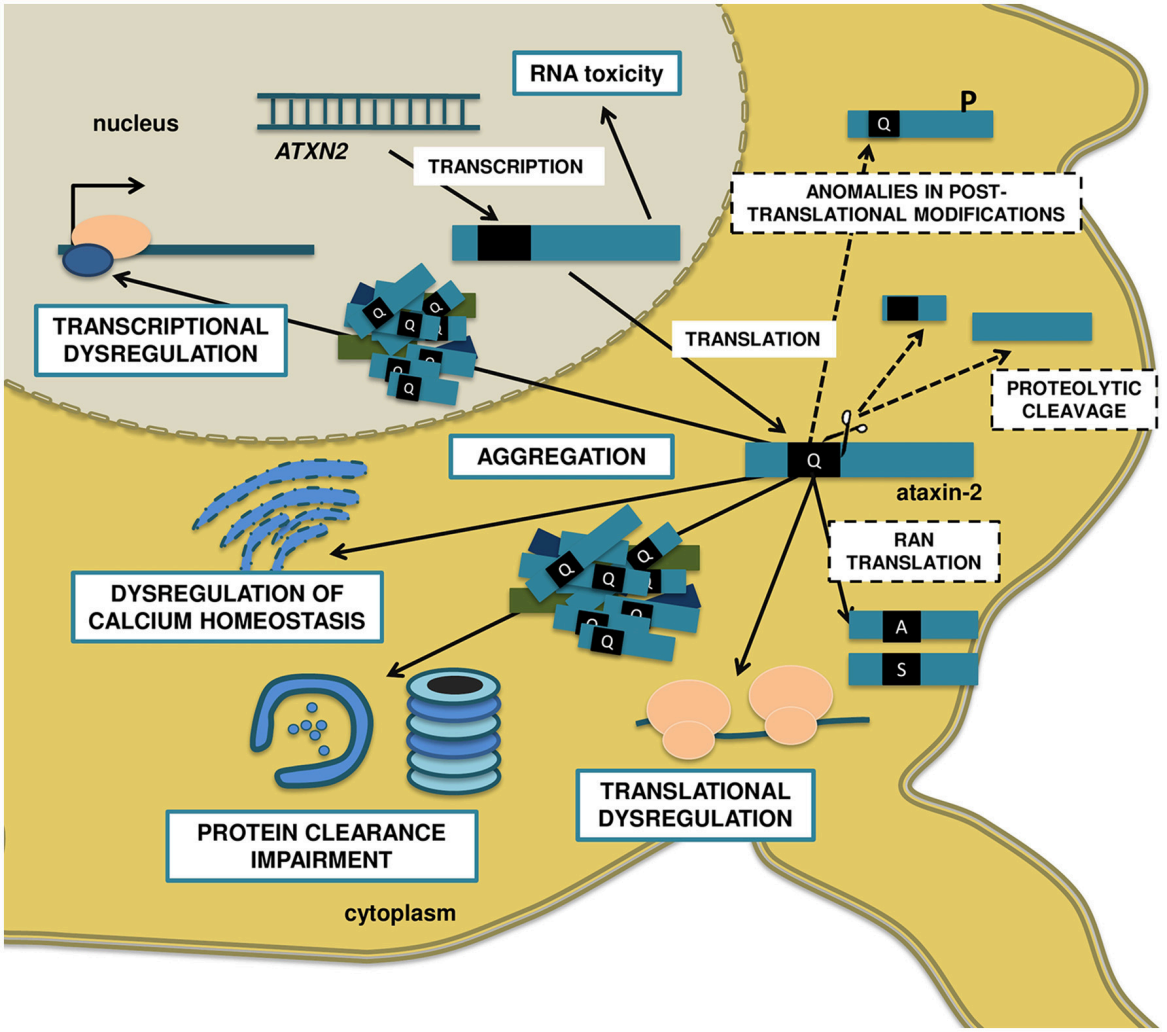
**Molecular mechanisms proposed to be involved in SCA2 pathogenesis**. The anti-sense transcription of the *ATXN2* gene gives origin to the repeat-expanded *ATXN2-AS*, with the ability to form hairpin structures and induce toxicity. The sense transcription encodes to the polyQ-expanded ataxin-2 protein, which assembles in insoluble, not always ubiquitinated cytoplasmic aggregates, and recruits other proteins like E3 ubiquitin ligases. Ultimately, the UPS might get overburden, disturbing the neuronal protein turnover and engaging in aberrant proteolytic cleavage, with the formation of N-terminal PolyQ-containing toxic fragments. Also, the mutated ataxin-2 decreases the ability of WT ataxin-2 to stabilize mRNAs and upregulate protein expression, resulting in transcriptional and translational dysregulations. On the other hand, the expanded polyQ protein binds to receptors in the ER and promotes a significant increase in intracellular calcium and, consequently, excitotoxicity, and enhanced LTD in cerebellar PCs. Finally, posttranslational modifications such as phosphorylation at specific residues might modulate the toxicity of ataxin-2, and RAN translation can result in additional polyalanine and/or polyserine toxic proteins. Blue, solid line boxes represent well-studied disease mechanisms, with extensive supporting evidence in SCA2. Black, dashed line boxes represent pathogenic mechanisms that are well established in other PolyQ disorders like HD and other SCAs, but whose relevance to SCA2 is still unclear.

The ATXN2 gene has 25 exons that encode for a protein with 1152 amino acid residues, when it possesses 22 glutamines (Scoles et al., 2012) and no sequence similarity to other polyQ proteins apart from the glutamine-rich domain (Magaña et al., 2013). The expansion of this domain above the disease threshold triggers a series of anomalies at the cellular level, presumably several years before the onset of the symptoms. In the following section, we summarize some of the proposed mechanisms for the pathophysiology of SCA2, which are illustrated in Figure 1.

### Aggregation

The aggregation of mutant polyQ proteins into cytoplasmic and/or intranuclear inclusions in neurons is a hallmark of these neurodegenerative diseases (Williams and Paulson, 2008; Bauer and Nukina, 2009; Takahashi et al., 2010). Nevertheless, the actual contribution of these aggregates to neurodegeneration has eluded the scientific community for many years, which tried to disclose their role as either causal or protective for the pathogenic process (Todd and Lim, 2013). Likewise, the mutant ataxin-2 aggregation and its role in SCA2 pathophysiology is also surrounded in controversy. A recent study tried to correlate the two main types of aggregates—granular cytoplasmic staining (GCS) and neuronal nuclear inclusions (NNI)—to the severity in neurodegeneration, with some interesting results (Seidel et al., 2016). Using post-mortem tissue from the brainstem of 5 SCA2 and 6 MJD patients, the authors found a significant positive correlation between the presence of GCS and a more severe neurodegeneration, while the NNI appeared to have a more protective role, at least in the MJD cohort. Also, Koyano et al. (2014) suggested that a cytoplasmic aggregation pattern of mutant ataxin-2 is prevalent in early stages of SCA2, whereas the presence of nuclear inclusions is related to a final stage. According to these and other studies (Ueda et al., 2014), the cytoplasmic ataxin-2 granules might be the first aggregated species in SCA2, actively contributing to pathogenesis by disrupting essential cell mechanisms. However, on an interesting note, the presence of aggregates in the cerebellar PCs remains quite controversial, with only a few reports of non-ubiquitinated cytoplasmic inclusions in mouse models (Huynh et al., 2000; Damrath et al., 2012).

### Protein recycling and proteolytic cleavage

Degradation of misfolded proteins typically occurs through two distinct pathways: the ubiquitin proteasome system (UPS) or autophagy. The dysfunction of the protein recycling pathways is a much debated topic in neurodegenerative diseases and has been extensively reviewed elsewhere (Berke and Paulson, 2003; Ross and Pickart, 2004; Matsuda and Tanaka, 2010; Takalo et al., 2013; Fecto et al., 2014; Cortes and La Spada, 2015). In SCA2, it was recently proven that mutant ataxin-2 can be selectively downregulated by FBXW8 (a subunit of an ubiquitin ligase complex), and that both FBXW8 and PARK2 (E3 ubiquitin ligase) are recruited to mutant ataxin-2 aggregates in cellular and animal models (Damrath et al., 2012; Halbach et al., 2015). This suggests a possible impairment in the UPS which, if perpetuated, might result in toxicity and neurodegeneration. The malfunction of the UPS can also result in increased cleavage of mutant proteins into smaller, more toxic fragments, which have been implicated in the pathogenesis of diverse neurodegenerative traits involving abnormal protein accumulation (Li et al., 2010; Matos et al., 2017). In SCA2, the artificial inhibition of the UPS originated a 70 KDa fragment in cells transfected with mutant ataxin-2, but not in those transfected with the WT form. Also, a 42 KDa fragment was detected in human post-mortem brain samples, reactive to the polyQ-specific 1C2 antibody (Huynh et al., 2000; Turnbull et al., 2004). Interestingly, this fragment (presumably N-terminal, given the position of the polyQ region) was detected both in a SCA2 patient and a cellular model of the disease, but not in control samples. This might suggest a link between mutant ataxin-2 proteolytic cleavage and SCA2 pathogenesis.

### Transcriptional and translational dysregulation

The most studied function of ataxin-2 is the regulation of translation and mRNA metabolism, as the very first efforts to characterize this protein accumulated compelling evidence toward this hypothesis. In fact, the first studies in yeast successively identified direct interactors of ataxin-2, like the poly(A)-binding protein 1 (PABP1) and the ataxin-2-binding protein 1 (A2BP1 or RBFOX1), that are known regulators of RNA metabolism (Mangus et al., 1998; Shibata et al., 2000). Since then, ataxin-2 has been shown to primarily localize in the Golgi apparatus (Huynh et al., 2003) and the endoplasmic reticulum (ER; van de Loo et al., 2009) and demonstrated to bind directly to the 3′UTRs of mRNAs, promoting their stability (Yokoshi et al., 2014). More importantly, these studies provided evidence that the PolyQ-expanded ataxin-2 caused cell death by disrupting the Golgi complex (Huynh et al., 2003) and actively downregulated the stabilizing effect of WT ataxin-2 in a PolyQ-length-dependent manner, decreasing its ability to upregulate protein expression (Yokoshi et al., 2014). So, in the global context of the disease, the expanded polyQ tract can affect the protein turnover of the cell, through a dysregulation of both synthesis (transcriptional and translational dysfunction) and degradation (UPS impairment). Considering the highly specialized and regulated functions of establishing and maintaining synaptic activity, neurons are particularly susceptible to alterations in protein turnover, which apparently happen in SCA2. This crucial role of WT ataxin-2 in maintaining mRNA stability and regulating translation might also explain the reason for ataxin-2 being associated with several other neurodegenerative diseases like ALS, SCA1, and MJD (Uchihara et al., 2001; Al-Ramahi et al., 2007; Lessing and Bonini, 2008; Elden et al., 2010; Nóbrega et al., 2015).

### Calcium dysregulation

The dysregulation of calcium signaling is found in many different neurodegenerative traits, and its role in the pathophysiology of these disorders has been the subject of many extensive reviews (Chopra and Shakkottai, 2014; Egorova et al., 2015; Meera et al., 2016). In SCA2 research, there is compelling evidence of abnormal calcium signaling, to which the PCs seem to be particularly susceptible, triggering the very first physical symptoms of the disease (Kasumu and Bezprozvanny, 2012). Indeed, in 2005 it was first reported that long, normal CAG repeats in the calcium voltage-gated channel subunit alpha 1A (CACNA1A) correlates with an earlier onset of symptoms in SCA2 patients (Pulst et al., 2005). Also, not only does ataxin-2 co-localize with the ER, which is the largest depository of intracellular calcium (van de Loo et al., 2009) but its mutated, expanded form binds to the C-terminal of the type 1 inositol 1,4,5-trisphosphate receptor (InsP_3_R1; Liu et al., 2009). This interaction significantly increases the release of intracellular calcium in primary PCs cultures from a SCA2 transgenic mouse model, and increases their sensitivity to glutamate-induced cell death (Liu et al., 2009). Interestingly, several *in vivo* experiments that targeted the activity of intracellular calcium receptors and calcium-activated potassium receptors alleviated many of the symptoms of these mice, establishing calcium dysregulation as a major mechanism of PC degeneration in SCA2 (Kasumu et al., 2012a,b; Liu et al., 2009). Finally, Kasumu and Bezprozvanny proposed that the increase in intracellular calcium mediated by expanded ataxin-2 intensifies cerebellar long-term depression (LTD) in an attempt to control excitotoxicity, which in turn triggers the first manifestations of ataxia (Kasumu and Bezprozvanny, 2012). A similar calcium dysregulation mechanism based on the same InsP_3_R1 receptor has also been proposed for HD and MJD, two of the most common polyQ disorders, suggesting that it might be a transversal mechanism in this group of diseases (Tang et al., 2003; Chen et al., 2008).

### RNA toxicity

For several years, the main focus of research in polyQ diseases has been the expanded polyQ tract of the affected proteins, and the different toxic effects that it exerts in neurons. However, recent studies have highlighted the contribution of non-coding RNA molecules, such as anti-sense and long non-coding transcripts, to the pathophysiology of polyQ and other neurodegenerative disorders (Moseley et al., 2006; Ladd et al., 2007; de Mezer et al., 2011). The mechanisms of RNA toxicity in neurodegeneration have been extensively reviewed elsewhere (Johnson et al., 2012; Salta and De Strooper, 2012; Tan et al., 2012) and include the sequestration of RNA-binding proteins, the production of mutant repeat RNA molecules by anti-sense transcription and the modulation of mutated genes by miRNAs. In SCA2, bidirectional transcription of the *ATXN2* gene has been recently reported, with the production of a neurotoxic anti-sense transcript (*ATXN2-AS*) as a consequence (Li et al., 2016). The supporting evidence is very robust, with the expanded CUG-repeat RNA being detected in the post-mortem *cerebellum* and cortex of SCA2 patients, human fibroblasts, human induced pluripotent stem cells (iPSCs), a BAC-transgenic mouse model and a lymphoblastoid cell line. Moreover, the transfection with non-translatable mutant *ATXN2-AS*, but not the WT *ATXN2-AS*, induced toxicity in SK-M-NC cells and primary mouse cortical neurons, presumably from the CUG-repeat tendency to form RNA hairpin structures (Li et al., 2016).

### Alternative disease mechanisms

The previously described mechanisms have substantial evidence to support their role in the pathophysiology of SCA2. However, other disease pathways have been well established for PolyQ diseases, including other SCAs, that are also worthy of mention despite their link to SCA2 remaining unclear. In fact, many different studies showed that phosphorylation and other posttranslational modifications of polyQ proteins might be implicated in different aspects of pathogenesis, either decreasing or enhancing neurodegeneration in HD, SCA1, and MJD (Humbert et al., 2002; Chen et al., 2003; Emamian et al., 2003; Luo et al., 2005; Pennuto et al., 2009; Warby et al., 2009; Matos et al., 2016). In SCA2, Turnbull and colleagues reported that ataxin-2 is indeed phosphorylated in SY5Y cells, presumably at the RXXSXP motif, similarly to ataxin-1 (Turnbull et al., 2004). Nevertheless, the impact of this modification in the disease pathogenesis remains elusive. Finally, a new mechanism for neurodegeneration was observed in SCA8, where it was found that a non-ATG-translation (RAN) of the CAG expansion resulted in polyserine and polyalanine proteins, apart from the polyQ protein (Zu et al., 2011). This work has caused a turmoil in the field of DNA repeat expansions disorders, with new non-ATG proteins being reported in several neurodegenerative traits, rendering the field far more complex than it was first considered (Cleary and Ranum, 2014). In SCA2, this phenomenon was also observed for the *ATXN2* gene, although in lower levels compared to canonical ATG translation and the study failed to detect polyserine and polyalanine proteins (Scoles et al., 2015).

## SCA2 mouse models

During the last two decades, a number of cellular and animal models of SCA2 have been developed to greatly contribute to our understanding of this disease. Indeed, although they are not the focus of this work, simple models like yeast, *C. elegans* and *D. melanogaster* were among the very first models used and have been crucial to infer on the ubiquitous functions of ataxin-2, such as: the embryonic development (Kiehl et al., 2000; Ciosk et al., 2004); the translation regulation and mRNA metabolism (Mangus et al., 1998; Satterfield and Pallanck, 2006); the regulation of circadian rhythms (Lim and Allada, 2013; Zhang et al., 2013); and modulating the formation of stress granules (Ralser et al., 2005). Likewise, the creation and characterization of two murine KO models of ATXN2 have provided precious new insights into the functions and pathways of this protein (Kiehl et al., 2006; Lastres-Becker et al., 2008a; Huynh et al., 2009; Fittschen et al., 2015; Halbach et al., 2016; Meierhofer et al., 2016) including a putative role in insulin resistance and obesity, which was validated in humans through Genome-wide association studies (Auburger et al., 2014). Finally, the development of iPSCs lines from SCA2 patients was recently reported, as well as CRISPR-Cas9 corrected isogenic iPSCs, to serve as suitable controls. These lines represent a unique opportunity to study the SCA2 disease mechanisms from actual patient-derived neurons (Xia et al., 2013; Marthaler et al., 2016a,b).

The purpose of this work is to present a comprehensive review on 5 mouse models of SCA2 that present behavioral characteristics resembling the human condition. In fact, the existing therapies for SCA2 rely basically on palliative care and non-specific medication to alleviate the major symptoms, which is inefficient to halt the disease progression. Thereby, in the last two decades, several authors created transgenic and Knock-in mice with the purpose of testing more effective therapeutic strategies that might be translated into the clinic. We aim to describe and compare each one of these models on the basis of their motor impairments, neuropathology and age of onset, and provide sustained examples of their usefulness in subsequent studies. To the best of our knowledge, this type of work has not been made for SCA2 and thereby it will constitute a valuable and important tool for researchers to choose the most adequate model addressing different scientific questions.

The following models will be discussed chronologically according to their publication year, consisting of 4 transgenic and 1 Knock-in (KI) mice: Q58 (Huynh et al., 2000); Q75 (Aguiar et al., 2006); Q42KI (Damrath et al., 2012); Q127 (Hansen et al., 2013); BAC-Q72 (Dansithong et al., 2015).

## Q58

This is the first report of a mouse model of SCA2, where the authors created a transgenic line that expresses a full length version of the human ATXN2, with 58 CAGs (Huynh et al., 2000). This mutant gene is regulated by a heterologous promoter (Purkinje cell protein 2—Pcp2) that directs the expression of the transgene to the PCs of the mice. The authors used the hybrid B6D2F1 as the genetic background for both the model and the control group, which expresses the cDNA of the human ataxin-2 with 22 glutamines (Q22). The most characterized mouse line was the Q58–11, which incorporated three copies of the human transgene, contrary to the Q22 lines, with just one copy.

The mice expressing the mutant ATXN2 exhibited clasping at 32 weeks of age, contrary to the control group (Q22). Also, the same animals showed a 19% reduction in the stride length at 16 weeks of age, when compared with the control and the WT. Likewise, the rotarod test showed a significant worse performance for the homozygous Q58 when comparing with the control group and the WT, with a smaller latency to fall (around 50%) at 16 weeks of age. These motor deficits were progressive, with both the homozygous and the heterozygous Q58 showing severe impairment at 26 weeks of age. There were no differences detected between the Q22 lines and the WT, in any of the tests. In a subsequent characterization done by Liu and co-workers (Liu et al., 2009), a general lack of balance and coordination was reported in the Q58 mice, as measured by the beamwalk test, when comparing with WT mice. These differences became significant at 32 weeks of age, including longer latencies to cross the beam and an increased number of foot slips, which worsened in a progressive manner with aging (Table 1).

**Table 1:** **Behavioral analysis of SCA2 mouse models**.

**Mouse model**	**Reduced body weight**	**Onset (weeks)**	**Impairments in rotarod**	**Onset (weeks)**	**Anomalies in footprint**	**Onset (weeks)**	**Clasping**	**Onset (weeks)**	**Impairments in the beamwalk**	**Onset (weeks)**
Q58	NA		Yes	16	Yes	16	Yes	32	Yes	32
Q75	NA		Yes	6	NA		NA		NA	
Q42KI	Yes	1	Yes	72	NE		NA		NA	
Q127	NA		Yes	8	NA		NA		NA	
BAC-Q72	Yes	8	Yes	16	NA		NA		NA	

*Behavioral hallmarks of the mouse models of SCA2. For each model, the symptoms/characteristics are included as existent (“Yes”), non-existent (“NE”), or Not available (“NA”), with the corresponding age of onset*.

The neuropathology in this model showed a decrease in the immunoreactivity with Calbindin-28K—a marker for neuronal dysfunction—at 4 weeks of age for the homozygous Q58. Similarly to the motor dysfunction, the loss of Calbindin-28K was progressive and worsened from 7 to 14 weeks. At 24–27 weeks of age, the authors also detected a significant loss of 50% in the number of PCs in the mutant lines, contrasting with the control lines that showed no signs of neuropathology at any time point. In a different work (Kasumu et al., 2012b), the Q58 transgenic mice were further characterized and an abnormal electrophysiological profile of these cells was reported at 24 weeks, worsening in a progressive manner with aging. This profile includes a decrease in the number of firing PCs and a reduced firing frequency of PCs in SCA2 mice, when compared with the WT (Table 2).

**Table 2:** **Neuropathology of SCA2 mouse models**.

**Model**	**Neurodegeneration**	**Onset (weeks)**	**Neuronal loss**	**Onset (weeks)**	**Ataxin-2 aggregates**	**Onset (weeks)**	**Morphological alterations**	**Onset (weeks)**	**Electrophysiological dysfunctions**	**Onset (weeks)**
Q58	Loss of calbindin-28K immunoreactivity	4	Decreased PCs number	24	No NIs. Cytoplasmic microaggregates		NA		Decrease in the number of firing PCs	24
									Reduced firing frequency of PCs	
Q75	Loss of calbindin-28K immunoreactivity	52	NA		NA		Loss of dendrites in PCs. Shrinkage of cell bodies in PCs	52	NA	
Q42KI	NE		NE		No NIs. Cytoplasmic insoluble aggregates	56	NA		NA	
Q127	NA		Decreased PCs number	40	Perinuclear aggregates	4	Loss of molecular layer thickness	12	Slower firing frequency of PCs	6
BAC-Q72	Loss of calbindin-28K immunoreactivity	24	NE		NA		Shrinkage of dendritic trees in PCs		NA	

*Neuropathological hallmarks of the mouse models of SCA2. For each model, a small description of its neuropathology and the corresponding age of onset is provided. NIs, Nuclear inclusions; NA, Not available; NE, Non-existent*.

## Q75

Another model was developed in 2006, when Aguiar et al. (2006) used the same methodology of pronuclear microinjection to create a mouse model with a human full-length version of ATXN2. The human gene was isolated from a Cuban patient with 75 CAG using RT-PCR and obtaining an amplicon of 4.477 Kb corresponding to the cDNA of the ataxin-2 isoform 1. This transgene was then expressed in the transgenic model under the regulation of the self-human SCA2 promoter. This approach allowed to obtain ubiquitous expression of the transgene across the entire body of the animal, as it was detected at the transcript level in the lungs, kidney, muscle tissue, brain and liver, and at the protein level in the cerebellum. The authors used the mouse hybrid strain B6D2F1 X OF1 as the genetic background for this model, and as a control strain for the behavioral tests, WT littermates. F066, that integrated one or two copies of the SCA2 transgene, was the most characterized line.

An ataxic behavior was observed in the Q75 mice when subjected to the rotarod assay. Indeed, a significant worse performance by the heterozygous transgenic animals was observed at 12 weeks, and by the homozygous at 6 weeks of age, when compared with the WT (Table 1).

Interestingly, the neuropathology revealed that, despite the ubiquitous expression throughout the body, only degeneration of PCs is reported, suggesting a somewhat targeted effect (Table 2).

## Q42KI

In 2012 it was published the first and only report so far of a knock-in mouse model of SCA2 (Damrath et al., 2012). In this work, Damrath and colleagues used the basic concept of the homologous recombination in embryonic stem cells to replace the normal murine atxn2 gene with a mutagenized version with 42 CAG repeats. This mutant form is under the control of the endogenous murine atxn2 promoter and was transmitted stably to the progeny for over nine consecutive generations. Moreover, the gene product was detected in the cortex and cerebellum at the transcript and protein level, demonstrating that the CAG expansion does not impair the atxn2 transcription and translation. The C57BL/6 mice strain was used as the genetic background and the WT littermates served as the controls for behavioral and neuropathological analysis.

By analyzing the phenotype of these Knock-ins, two interesting conclusions were drawn. First, as early as 1 week of age was enough to detect a significant 19% reduction in the weight of these animals as compared with the WT, a difference that remained for their entire life. Secondly, the mutant mice exhibited a very mild ataxic behavior: no differences were found in the open field, grip or footprint analysis between the Knock-ins and the WT. As for the accelerating rotarod, the differences became significant only at 72 weeks of age, for the homozygous mice (Table 1). On its turn, the heterozygous did not show any impairment at all when compared with the WT, at any time point analyzed.

The neuropathological assessment also revealed very mild and late-onset alterations, consistent with the behavioral results. In fact, the only perceptible alterations were cytoplasmic ataxin-2-containing insoluble aggregates, identified in the PCs of the Knock-in mice. These aggregates first became detectable at 56 weeks of age, and then at 96 weeks in much higher number. There were no signs of neuronal loss, neurodegeneration or nuclear inclusion bodies (Table 2).

## Q127

Another transgenic mouse model was created in Hansen et al. (2013) with a slightly different objective and thereby a distinct approach. The purpose was to evaluate the electrophysiological and gene expression changes in PCs in SCA2, and to correlate those variations with the onset of the motor phenotype. With this objective in mind, Hansen and co-workers created a model mice with 127 CAG repeats under the Pcp2 promoter to try and obtain a strong and targeted degeneration in the PCs as well as a robust ataxic phenotype. The authors used the mouse strain B6D2F1 as the genetic background for this model and, as a control strain for the behavioral tests, WT littermates were used.

As early as 8 weeks was enough to show a motor dysfunction in the accelerating rotarod in the transgenic mice, which worsened in a progressive manner until 36 weeks of age. By this time point, the latency to fall of the mutant animals showed a significant reduction of 50% when compared with the WT littermates (Table 1).

The neuropathology of the Q127 mice was also altered, with the presence of ataxin-2 aggregates in the PCs at 4 weeks, and increasing in number in subsequent time points. Another important feature was the decrease in the molecular layer thickness, which became significant at 12 weeks of age and progressively aggravated, becoming over 60% reduced comparing with the WT littermates. Neuronal loss was also observed in the transgenic animals, at 40 weeks of age, with a reduction in the number of PCs. Finally, the electrophysiological profile of these cells also showed differences in the mutant animals, namely a slower firing frequency that was significant at 6 weeks and worsened with age (Table 2).

## BAC-Q72

Finally, in 2015, Dansithong and co-workers developed the most recent transgenic mouse model of SCA2 available, with 72 CAG repeats (Dansithong et al., 2015). This model was created with Bacterial Artificial Chromosomes (BAC), which are DNA constructs based on a functional fertility plasmid (or F-plasmid). In recent years, these large-insert DNA clones have been used to create transgenic animals mainly due to its capacity to include an entire gene sequence, including non-coding regions (Heintz, 2001; Van Keuren et al., 2009). As for the genetic background, the authors used the FVB mouse strain for both the model and the control group, which contains the entire gene of the human ATXN2 with 22 CAGs (BAC-Q22). Also, the BAC-Q72 and the BAC-Q22 integrated 4 and 10 copies of the corresponding transgenes, respectively, and they are both regulated by the endogenous human promoter. In conclusion, this strategy allowed obtaining ubiquitous expression of the transgenes across the entire body of the animal, as it was detected at the transcript level in the heart, liver, and the entire central nervous system and at the protein level in the cerebellum. The most characterized mutant line was the BAC-ATXN2-Q72.

Similarly to the Q42KI, both the BAC-Q72 and the BAC-Q22 also weighted significantly less than the WT, starting at 8 weeks of age and progressing until a 30% reduction when comparing to the WT littermates. The transgenic with the mutant ataxin-2 suffered impairments in the motor coordination, showing a decreased latency to fall in the accelerating rotatod, which started at 16 weeks of age and progressively worsened until the 36 weeks (Table 1).

The neuropathological analysis also showed dysfunctions in these BAC-Q72 mice, at 24 weeks, with shrinking of PCs dendritic trees and loss of the calbindin and Pcp2 proteins in the cerebellum (Table 2).

## Discussion

The development of mouse models that mimic the SCA2 phenotype was needed to: (a) facilitate the testing of new therapeutic strategies, and (b) allow the study of the disease progression in an animal that is biologically close to humans. So far, 5 different mouse models have been created that assume behavioral signs/symptoms found in human patients. Considering the number and severity of the symptoms, their age of onset, the neuropathological progression and the design strategy itself, each one of these models can prove to be quite useful when addressing different scientific questions. In Table 3, we summarized the key features of each mice that we consider to be either advantageous or not for different researching purposes, putting together a global view of the available murine models of SCA2.

**Table 3:** **The major advantages and downsides of SCA2 mouse models**.

**Mouse models**	**Representative line**	**Advantages**	**Disadvantages**
Q58	Q58-11	Robust, well-characterized behavioral impairments and neuropathology;	Unable to study other brain regions: targeted expression to PCs;
		Suitable to study PCs dysfunction;	Non-physiological expression levels of ataxin-2—overexpression;
		Practical for research into new therapeutic strategies;	Pronuclear injection: random integration;
Q75	F066	Ubiquitous expression of mutant ataxin-2;	Pronuclear injection: random integration;
		Integrated only 1 or 2 copies of the transgene;	Lack of control transgenic line;
		Early onset of symptoms and robust motor impairment—practical in testing for new therapies;	No other symptom besides motor incoordination;
Q42 KI	CAG42	Very faithful to the human condition in terms of physiological expression levels and age of onset;	Mild ataxia phenotype and neuropathology with late onset of symptoms;
		Ideal to study early differential gene expression levels;	Unpractical for testing new therapies that alleviate symptoms;
		Potential to find disease biomarkers;	
		Targeted insertion;	
Q127	ATXN2^Q127^	Very robust impairments in behavior and neuropathology with early onset of symptoms;	Unable to study other brain regions: targeted expression to PCs;
		Suitable to study electrophysiological dysfunctions in PCs and testing new therapies;	Decreased faithfulness to the human disease;
			Pronuclear injection: random integration;
BAC-Q72	BAC-ATXN2-Q72	The entire gene (introns included) was inserted into the mouse genome: suitable to study RNA-related mechanisms;	No other symptoms besides motor incoordination;
		Ubiquitous expression of mutant ataxin-2;	Mild neuropathology;

*The major advantages and disadvantages of SCA2 mouse models. For each mutant mouse line, the most notorious upsides and downsides are given to facilitate the choice of the most adequate model addressing different biological questions*.

The Q58 transgenic mice have a very well characterized and progressive ataxic phenotype and neuropathology as its greatest advantage. In fact, these mice exhibit not only a strong motor impairment in the rotarod, but they are also the only described model with anomalies in the footprint, beamwalk and clasping when comparing with a control Q22 group (Table 1). These behavioral tests are commonly considered by experts (Brooks and Dunnett, 2009) to be strong indicators of motor incoordination, lack of balance and an abnormal gait, which are key features of the cerebellar ataxia described in human SCA2 patients (Pulst, 2015). Besides that, they have a marked neuropathological phenotype, with clear signs of neuronal dysfunction, neuronal loss and cytoplasmic polyQ aggregates in PCs, all of which were corroborated in post-mortem human tissue (Estrada et al., 1999; Koyano et al., 1999; Huynh et al., 2000; Ishida et al., 2011). Interestingly, electrophysiological impairments in Q58 PCs have also been reported, nearly concomitant with the onset of behavioral anomalies, suggesting that the burst firing of these neurons is directly linked with the ataxic symptoms (Kasumu et al., 2012b). These behavioral and neuropathological traits are common to several SCAs, as it was evidenced by similar results in mouse models of SCA1 (Burright et al., 1995), MJD (Chou et al., 2008; Torashima et al., 2008), or SCA7 (Garden et al., 2002). The diversity of the Q58 phenotype and its extensive characterization make this mouse model quite practical to study the effect of new therapeutic strategies in different aspects of the SCA2 phenotype, as it was demonstrated already by some interesting works: Liu et al. (2009) treated the Q58 mice with a pharmacological inhibitor of the Ryanodine receptor, successfully improving the performance of the mutant animals in the beamwalk and the rotarod, and establishing this receptor as a potential therapeutic target for SCA2; Chang et al. (2011), rescued some of the Q58 motor symptoms by injecting these mice intravenously with human mesenchymal stem cells; Kasumu et al. (2012a) used a selective modulator of the SK2/3 channels, administered orally, to alleviate the motor dysfunctions of this model, achieving a significant improvement in the beamwalk and accelerated rotarod; on a different work, Kasumu et al. (2012b) relied on a phosphatase, delivered by adeno-associated viruses, to inhibit the 1,4,5-triphosphate receptor-mediated calcium release. This strategy was successful in preventing the PC dysfunction and rescuing some of the motor impairments of transgenic mice. Nevertheless, there are some issues worth of notice in this model. First, it uses a heterologous promoter that, unlike the human patients that have expression of mutant ATXN2 across the whole body, directs the expression exclusively to the PCs. This feature, although useful for the study of cerebellar dysfunctions like protein aggregation in PCs, hamper the analysis of other brain regions and non-cerebellar symptoms such as Parkinsonism and ALS-related degeneration of motor neurons (Shan et al., 2001; Furtado et al., 2004; Elden et al., 2010). Second, the method of pronuclear injection by which this model was generated results in the integration of the transgene in a random location of the genome, which might be disrupting the function of endogenous genes. Finally, the three Q58 founder mice integrated two, three or four copies of the transgene, leading to non-physiological levels of ataxin-2 expression.

Unlike the Q58, the Q75 model exhibits expression of the transgene across the whole body, as it was detected at the transcript level in the lungs, kidney, brain, liver, and skeletal muscle of the mice, and at the protein level in the *cerebellum*. This renders the model more faithful to the human condition, since ataxin-2 is ubiquitously expressed throughout the entire human body (Imbert et al., 1996; Pulst et al., 1996; Sanpei et al., 1996). This feature should allow to study different brain regions and their contributions to the pathophysiology of the disease, although no reports were found of such studies. Moreover, the transgenic mice display a severe motor impairment, with an earlier onset of those symptoms than any other model, which makes them less expensive and time-consuming to test new therapies and evaluate its effect in an ataxic phenotype. The major disadvantages lie with the method of pronuclear injection that promotes random integration, and also to the lack of a transgenic animal line with wild-type ATXN2, to serve as a suitable control. Although the motor incoordination is quite severe, there is no information available of any other motor behavior (Table 1) and the neuropathology is not as well-characterized as in the Q127 or in the Q58 (Table 2).

Contrasting with every other model, the Q42KI mice present a faint and late-onset phenotype, probably due to the reduced number of CAGs. Indeed, although these mice display a reduced body weight starting in the first week of age, the behavioral symptoms appear at 72 weeks, with a mild impairment in motor coordination, and only for the homozygous animals (Table 1). Likewise, the only neuropathological hallmarks detected were the presence of cytoplasmic ataxin-2 aggregates, at 56 weeks (Table 2). An early-onset and fast-progressive ataxia are usually preferable when testing new therapeutic strategies or drugs, for which reason the Q42KI are impractical for this purpose. Also, the heterozygous Q42KI did not show any motor impairments when compared with the WT, contrary to what happens in human SCA2 patients. Notwithstanding this, the faithfulness of these mice to the human condition is unparalleled by any other model available: physiological levels of expression, targeted insertion, and a late onset of the symptoms. In fact, transgenic mice represent an overexpression paradigm that explains the earlier onset and severer phenotype, but their modeling is limited to the very last stages of SCA2, disregarding the long pre-symptomatic stage of the human condition. Thereby, the Q42KI surges as the best model to study the early molecular mechanisms involved in the pathology of SCA2. The authors of this work did exactly that, by performing a genome-wide transcriptomic analysis in the cerebellum, brain stem, and liver of the Knock-in and the WT mice, and identifying one gene—Cyp4a14, involved in cholesterol biosynthesis—differentially expressed in the liver at 24 weeks of age. In a subsequent study, Halbach et al. (2016) investigated the cerebellar transcriptome of these Q42KI, finding downregulated expression levels of two genes involved in calcium homeostasis: Atp2a2 and Itpr1, which were also downregulated in SCA2 KO mice (Lastres-Becker et al., 2008a). These works might pave the way to identify new disease biomarkers and also strengthen the hypothesis that deranged calcium signaling is a key mechanism in the PCs degeneration found in the transgenic models (Liu et al., 2009).

The Q127 transgenic shows a very robust, early-onset, and well-characterized phenotype. Indeed, the expression of *ATXN2* containing a high number of CAG repeats with a PC-specific promoter allowed to obtain a severe dysfunction in the motor coordination of these mice, which can be observed as early as at 8 weeks of age. Likewise, several aspects of the neuropathology have been well studied, with a significant decrease in the number of PCs, perinuclear aggregates and a decrease in the molecular layer thickness, assembling a range of features that are frequently found in human patient's data (Estrada et al., 1999; Koyano et al., 1999; Huynh et al., 2000). Furthermore, the diversity of the phenotype and its early-onset make this model quite practical for testing new therapeutic strategies and assessing its effect in ataxia. The Q127 mice also offer a good opportunity to study the SCA2-related neuronal dysfunction of PCs with more detail, as the authors reported severe anomalies in the electrophysiological profile of these cells, with a 75% reduction in the firing frequency compared to WT mice. The biggest disadvantage of this model, to our understanding, lies with aspects that confer a lower biological relevance when comparing with the Q42KI or the BAC-Q72. Indeed, the most frequent disease alleles of SCA2 contain between 37 and 41 CAG repeats (Geschwind et al., 1997; Pulst, 2015) with only exceptional cases reporting >100 CAG repeat alleles (Babovic-Vuksanovic et al., 1998; Paciorkowski et al., 2011). Together with the targeted expression to the PCs, these transgenic mice are not as faithful to the human condition and actually model a very late stage of SCA2. The likely insertional mutagenesis derived from the method of pronuclear injection and the lack of a transgenic animal line with wild-type ATXN2, to serve as a suitable control, might also be considered as downsides to this model.

Finally, the most recent transgenic model of SCA2 presents a new approach, which is the use of the Bacterial Artificial Chromosomes to insert the mutant ATXN2 transgene. This strategy has been used to create some very robust models of disease (Gray et al., 2008; Li et al., 2009) and its advantages include the insertion of the entire gene sequence into the host genome. This feature allows to study the contribution of normal splicing events and the regulation by non-coding regions of ATXN2 to the pathophysiology of the disease, as recently demonstrated by Li et al. (2016). In this work, the authors successfully identified the *ATXN2-AS* transcript in cortex and *cerebellum* of the BAC-Q72, a non-coding and expanded RNA with toxic properties whose expression was corroborated in numerous sources of human samples. This would have been impossible in any other transgenic mouse model that uses cDNA to express the mutant ataxin-2. In fact, considering the increasing relevance given to RNA-related mechanisms of toxicity in neurodegenerative diseases, the BAC-Q72 is the most adequate model to study these phenomena in SCA2 (Johnson et al., 2012; Tan et al., 2012; Cleary and Ranum, 2013; Ramaswami et al., 2013). Besides this, the mice also displayed a moderate ataxic phenotype at 16 weeks, with impairments in the accelerated rotarod, which worsened in a progressive manner. As disadvantages, we refer to the mild neuropathology, consisting in a slight dysfunction of PCs, which make it less appealing to evaluate the effect of potential therapies in disease progression.

To summarize, all the mouse models of SCA2 have advantages and disadvantages, but they have all proven very useful to the understanding of this terrible neurodegenerative condition. Indeed, the Q42KI allows studying the very early molecular mechanisms of pathogenesis with the potential to identify new disease biomarkers, while the transgenic models (Q58, Q75, and Q127) might lack some physiological relevance, but possess robust phenotypes and early onset of symptoms. This makes them highly practical for testing new therapeutic strategies, allowing its assessment in various aspects of behavior and neuropathology in a less expensive and time-consuming manner. Finally, the most recent transgenic mice (BAC-Q72) show promise in studying new emerging RNA-related mechanisms of SCA2 pathology. On a final note, *ATXN2* has been shown to take part in various other neurodegenerative diseases besides SCA2, such as ALS, SCA1, MJD, and PD, revealing an intrinsic cascade of pathways regulated by this gene (Al-Ramahi et al., 2007; Lessing and Bonini, 2008; Elden et al., 2010; Nóbrega et al., 2015; Nkiliza et al., 2016; Sen et al., 2016). The available mouse models of SCA2 are insufficient to explore these new lines of research, highlighting the urgency of developing new ones that model the neurodegeneration of dopaminergic midbrain neurons and motor neurons, affected in PD and ALS.

## Author contributions

CN—idealized the paper, co-wrote the manuscript, and made a scientific review; JA—made the bibliographic review and co-wrote the paper; LM—co-wrote the paper; LP—made a scientific review of the manuscript.

## Funding

The work in the author's laboratories is funded by Foundation for Science and Technology, Portugal, by the French Muscular Dystrophy Association, France, and by the National Ataxia Foundation, USA.

### Conflict of interest statement

The authors declare that the research was conducted in the absence of any commercial or financial relationships that could be construed as a potential conflict of interest.

## Abbreviations

SCA2: Spinocerebellar ataxia type 2
PolyQ: Polyglutamine
HD: Huntington's disease
SBMA: Spinal bulbar muscular atrophy
SCAs: Spinocerebellar ataxias
SCA6: Spinocerebellar ataxia type 6
SCA1: Spinocerebellar ataxia type 1
MJD: Machado-Joseph disease
SCA3: Spinocerebellar ataxia type 3
PCs: Purkinje cells
GCS: Granular cytoplasmic staining
NNI: Neuronal nuclear inclusions
ALS: Amyotrophic lateral sclerosis
CACNA1A: Calcium voltage-gated channel subunit alpha 1A
InsP_3_R1, Type 1 inositol 1, 4: 5-trisphosphate receptor
LTD: Long-term depression
iPSCs: Induced pluripotent stem cells
RAN: Repeat-associated non-ATG translation
KO: Knock-out
KI: Knock-in
Pcp2: Purkinje cell protein 2
cDNA: Complementary DNA
BAC: Bacterial artificial chromosome
F-plasmid: Functional fertility plasmid
SCA7: Spinocerebellar ataxia type 7
SK2/3, Small-conductance: Ca^2+^-activated K^+^channels Type 2/3
PD: Parkinson's disease.
